# Leukocytospermia and sperm preparation - a flow cytometric study

**DOI:** 10.1186/1477-7827-7-128

**Published:** 2009-11-19

**Authors:** Giuseppe Ricci, Sandra Perticarari, Rita Boscolo, Roberto Simeone, Monica Martinelli, Leo Fischer-Tamaro, Secondo Guaschino, Gianni Presani

**Affiliations:** 1 Assisted Reproduction Unit, Department of Obstetrics and Gynaecology, Institute for Maternal and Child Health IRCCS Burlo Garofolo and University of Trieste, Via dell'Istria 65/1, Trieste 34137, Italy; 2 Clinical Analysis Unit, Department of Laboratory Medicine, Institute for Maternal and Child Health IRCCS Burlo Garofolo, Via dell'Istria 65/1, Trieste 34137, Italy

## Abstract

**Background:**

Leukocytes represent the predominant source of reactive oxygen species both in seminal plasma and in sperm suspensions and have been demonstrated to negatively influence sperm function and fertilization rate in assisted reproduction procedures. Peroxidase test is the standard method recommended by WHO to detect semen leukocytes but it may be inaccurate. The aims of this study were (i) to compare the efficiency of swim-up and density-gradient centrifugation techniques in removing seminal leukocytes, (ii) to examine the effect of leukocytes on sperm preparation, and (iii) to compare flow cytometry and peroxidase test in determining leukocyte concentration in semen using a multiparameter flow cytometric method.

**Methods:**

Semen samples from 126 male partners of couples undergoing infertility investigations were analyzed for leukocytospermia using standard optical microscopy and flow cytometry. Sixty-nine out of 126 samples were also processed using simultaneously the swim-up and density-gradient centrifugation techniques. A multiparameter flow cytometric analysis to assess simultaneously sperm concentration, sperm viability, sperm apoptosis, and leukocyte concentration was carried out on neat and prepared sperm.

**Results:**

Both sperm preparation methods removed most seminal leukocytes. However, the concentration of leukocytes was significantly lower after swim-up compared to that after density-gradient centrifugation preparation. Leukocytes concentration, either initial or in prepared fractions, was not correlated with sperm parameters (optical microscopy and flow cytometry parameters) after semen processing. There was no correlation between leukocyte concentration in the ejaculate and sperm recovery rate, whereas a significant correlation was found between the concentration of the residual leukocytes in prepared fractions and viable sperm recovery rate. Although the overall concordance between the flow cytometry and the optical microscopy was satisfactory, the sensitivity of peroxidase test for the detection of leukocytospermia resulted low.

**Conclusion:**

Seminal leukocytes do not seem to influence sperm preparation results. However, for assisted conception, semen samples containing leukocytes should be processed using swim-up method. Although peroxidase-test is recommended by WHO as the standard method for determining semen leukocytes, it should not be used in clinical research study.

## Background

Sperm preparation methods should eliminate dead spermatozoa and other cells, including bacteria and leukocytes [1]. Seminal leukocytes represent the predominant source of reactive oxygen species (ROS) in seminal plasma [2-4] and have been demonstrated to be involved in retroviruses transmission [5], whereas their role in the male infertility etiology is still controversial [6]. Several studies have investigated the relationship between leukocytospermia and semen parameters [7-15] but very few data are available about the relationship between leukocytospermia and sperm preparation [16]. It has been demonstrated that even sperm fractions prepared for assisted reproduction treatment are frequently contaminated with leukocytes [17,18] and that leukocytes are the main source of ROS in sperm suspensions [2,8,19-21]. Reactive oxygen species, produced by leukocytes, can penetrate the sperm plasma membrane and induce intrinsic ROS production by sperm [13]. It has been shown that intrinsic ROS production is correlated with sperm DNA fragmentation [13]. Although leukocytes produce ROS at least thousand times more than sperm [3], the intrinsic ROS production may represent an important variable in terms of fertility potential [6,13]. Sperm damage from ROS can occur when seminal plasma is removed during sperm preparation [22]. The presence of one activated leukocyte per 20000 sperm would produce a detectable amount of ROS [23], so, even a very low number of leukocytes in the sperm suspension may influence the integrity of sperm and, consequently, the outcome of assisted reproduction treatment [6]. Therefore, it is of paramount importance to use a very sensitive method to detect seminal leukocyte. Peroxidase test is the standard method recommended by WHO [24] to detect semen leukocytes but it may be inaccurate [25,26].

Swim-up and density-gradient centrifugation remain the most common methods used for the isolation of functionally normal spermatozoa [1]. Swim-up should, theoretically, provide a sperm suspension with a lower level of leukocyte contamination compared to that obtained after density-gradient centrifugation. However, the two methods have never been compared. Recently, we have introduced a novel multiparameter flow cytometry method that offers the possibility of a simultaneous, simple, rapid, and accurate assessment of several semen parameters, including functional parameters, such as viability, necrosis, and apoptosis [27]. This method allows also performing a highly precise count of seminal leukocytes.

The objectives of our study were (i) to compare the efficiency of swim-up and density-gradient centrifugation techniques in the removal of seminal leukocytes; (ii) to examine the effect of leukocytes on sperm preparation; and (iii) to compare flow cytometry and peroxidase test in determining leukocyte concentration in semen.

## Methods

### Semen samples

Semen samples were obtained from 126 men (mean age 36.4 ± 6.4 years) undergoing routine infertility investigations at the Assisted Reproduction Unit of the Institute for Maternal and Child Health IRCCS Burlo Garofolo and University of Trieste. All subjects were the partners of women who failed to conceive after 24 months of unprotected intercourse. All subjects were asymptomatic for genitourinary infections. Semen samples were collected by masturbation into sterile containers after 3-4 days of sexual abstinence. After complete liquefaction, routine semen analysis was performed using a light microscope according to World Health Organization guidelines [24].

A leukocyte count was carried out by using standard peroxidase test, as described in the WHO laboratory manual [24]. Leukocytospermia was defined as the presence of > 1 × 10^6 ^leukocytes per milliliter of semen [24]. From each ejaculate an aliquot of semen was taken for the multiparameter flow cytometric analysis. Sixty-nine out of 126 samples were also processed using simultaneously the swim-up and density-gradient centrifugation techniques. The multiparameter flow cytometric analysis was carried out also on prepared sperm. The study was approved by the Institutional Review Board.

### Density-gradient centrifugation

The sperm preparation was done using 40-80% double density gradient (PureSperm, Nidacon International, AB, Goteborg, Sweden). Media were brought to 37°C temperature. Using a sterile pipette, 0.5 ml of liquefied semen sample was placed on top of the upper layer into a 5-ml Falcon conical tube (Becton Dickinson Labware, Meylan, France). The tube was centrifuged at 300 × g for 20 minutes. The supernatant was then removed and the pellet was suspended in a volume of 1 ml of medium and again centrifuged at 500 × g for 10 minutes. The pellet was resuspended in a volume of 0.5 ml of medium. An aliquot was examined for sperm concentration, sperm motility, and leukocytes concentration. Another aliquot was used for the multiparameter flow cytometric analysis.

### Swim-up

An aliquot of 0.5 ml of semen was washed with 1 ml of medium (Quinn's Advantage Medium w/HEPES, SAGE BioPharma™, Bedminster, NJ, USA, supplemented with 0.5% human serum albumin, SAGE Assisted Reproduction Products™, CooperSurgical, Trumbull, CT, USA) into a 5-ml Falcon conical tube (Becton Dickinson Labware, Meylan, France) and then centrifuged at 300 × g for 10 min. The supernatant was discarded and the pellet was resuspended in 0.5 ml of medium. Then, 0.5 ml of medium was gently layered on sperm suspension and the tube was inclinated at an angle of 45 degrees and incubated at 37° for at least 45 min. The tube was gently set upright and the upper interface was then gently aspirated with a Pasteur pipette. An aliquot was examined for sperm concentration, sperm motility, and leukocytes concentration. Another aliquot was used for the multiparameter flow cytometric analysis.

### Multiparameter flow cytometric analysis

A multiparameter flow cytometric analysis to assess simultaneously sperm concentration, sperm viability, sperm apoptosis, CD45 positive cell (leukocyte) concentration was carried out, as previously described [27], with minor modifications. Sperm before and after semen preparation were analysed. Briefly, 100 μl of neat semen or prepared sperm were stained for 20 min in the dark at room temperature using 2 μl of a 10-μM solution of Syto 16 Green-Fluorescent nucleic acid stain from Molecular Probes (Eugene, Oregon, USA) (final concentration 200 nM), 10 μl of 7-amino-actinomycin D (7-AAD Via-Probe, BD Pharmingen, San Diego, CA, USA), and 10 μl of allophycocyanin conjugated anti-CD45 monoclonal antibody (mAb).

The sperm in neat semen were counted and diluted in medium to reach approximately the same concentration of prepared sperm (1-10 × 10^6^/ml). A Flow-Count™ fluorospheres vial (Beckmann-Coulter, Fullerton, CA, USA, lot 754863), at a concentration of 1016 beads/μl, was gently mixed for 10-12 sec, and 100 μl of fluorospheres were accurately pipetted (precision reverse pipetting with wet tip) before analysis. After the incubation period, 1 ml of cold phosphate-buffered saline (PBS) was added to each tube, and the samples were analysed by flow cytometry.

Flow cytometric analysis was performed by using a FACSCalibur four-colour (Becton Dickinson, San Josè, CA, USA) equipped with a 488-nm argon laser with 530-nm (FL1), 585-nm (FL2) and 670-nm (FL3) band-pass fluorescence filters and a 635-nm red diode laser with a 661-nm band-pass filter (FL4). One hundred thousand events were collected in list mode and analysed with CELLQuest Pro software. A gating strategy was used to allow the identification of viable, dead and apoptotic sperm, as well as of CD45 positive cells and of fluorospheres.

**Figure 1:**
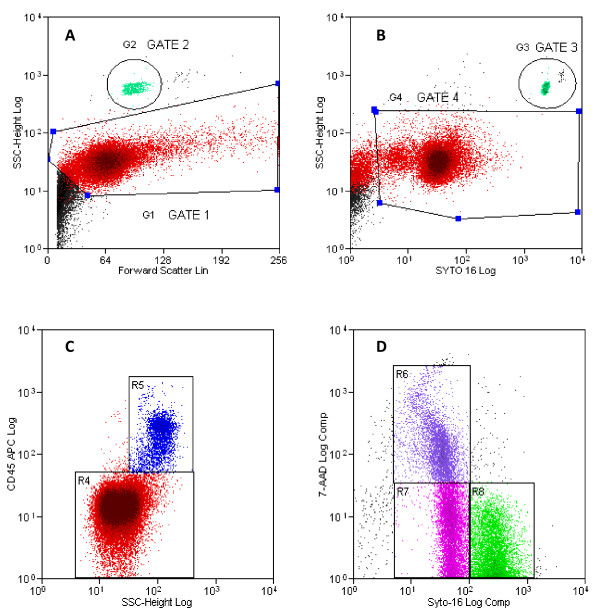
**Flow cytometric gating strategy for specific identification of viable, dead and apoptotic sperm, and leukocytes**. Plot A shows semen population gated on the basis of scattering measurements (forward-angle scatter versus side-angle scatter); Gate 1 was made to identify the semen cell (red) and exclude the debris (black). Plot B shows the gate G4, made on Syto 16 versus SSC parameters to identify the nucleated cells stained with Syto 16. Two gates G2 (plot A) and G3 (plot B), in green, were set to accurately identify bead population. Plot C represents the events gated in G4 that were analyzed in FL4 versus SSC to identify the CD45 positive cells (leukocytes) (R5) and the CD45 negative cells (sperm) (R4). Plot D shows the events gated in R4 (sperm) that was analyzed in another cytogram, Syto 16 versus 7-AAD, to identify viable (R8) apoptotic (R7) and dead sperm (R6).

A first gate (G1) was set on the entire semen cell population on the basis of scattering measurements (forward-angle scatter vs. side-angle scatter) (Figure 1A). Since the gate based on side-angle and forward-angle scatters could also include cells or debris with similar sizes and granularities as sperm, the method based on Syto 16 staining [27] was applied to allow a more precise identification of semen cell population; in order to exclude the Syto 16 negative events, another gate G4 was made on FL1 versus SSC (Figure 1B). Events gated in G4 included both syto-16^low ^and syto-16^high ^sperm and leukocytes. Then, they were analyzed in a plot of FL4 versus SSC (Figure 1C) to identify the CD45 positive cells (leukocytes) (R5, Figure 1C) and the CD45 negative cells (sperm) (R4, Figure 1C). Gated population in R4 (Figure 1C), representing only sperm, was then analyzed in another cytogram, Syto 16 (FL1) versus 7-AAD (FL3), where the Syto 16 population displays different expression of 7-AAD (Figure 1D). By using this gating strategy it was possible to distinguish between viable, apoptotic, and dead sperm (Figure 1D). Syto 16^high^/7-AAD^neg ^sperm were defined as viable (R8, Figure 1D), Syto 16^low^/7-AAD^neg ^sperm as apoptotic (R7, Figure 1D), Syto 16^low^/7-AAD^pos ^sperm as necrotic (R6, Figure 1D). Two gates (G2, G3) were set on FL1 versus SSC and on FSC versus SSC to identify accurately the fluorosphere population (Figure 1B). The left side marker used to discriminate the Syto 16 positive from the Syto 16 negative cells, was determined using a negative control containing only medium, without stain. The plots are not shown for an easier graphic presentation. Sperm and CD45 positive cells concentrations were calculated according to the formula:

Concentration of Fluorospheres indicates the number of Fluorospheres per μg/l (known concentration) as given by the manufacturer, referred to the volume pipetted per sample.

### Statistical Analysis

Comparisons between neat semen and prepared sperm from the same ejaculate were performed using the Wilcoxon matched pairs test, as well as between the two sperm preparation methods. The relationship between semen analysis parameters and flow cytometry results were analysed using the Spearman rank correlation test. Concerning leukocyte detection, the concordance between peroxidase test and flow cytometry results was calculated by overall percentage agreement and by Cohen's Kappa statistics [28]. The relationship between the Kappa value and the level of agreement was suggested by Landis and Koch [29]: a kappa value of 0.00-0.20 represents slight agreement; 0.21-0.40, fair agreement; 0.41-0.60, moderate agreement; 0.61-0.80, substantial agreement; and 0.81-1.00, almost perfect agreement. All statistical tests were two-sided and a P value of < 0.05 was considered to be statistically significant.

Statistical analysis was carried out using GraphPad Prism version 5.00 (GraphPad Software, San Diego, CA, USA) and Statistica Version 8 (StatSoft, Tulsa, OK, USA).

## Results

The summary statistics of the semen parameters analyzed in the study are reported in Table 1. Twenty-three subjects were normozoospermic, 18 were asthenozoospermic, 4 were oligoasthenozoospermic, 12 were asthenoteratozoospermic, and 12 were oligoasthenoteratozoospermic.

**Table 1:** Summary statistics of the sperm parameters analyzed in the study.

	Mean ± SEM	Median
Concentration (× 10^6^/mL)	77 ± 9.3	58.0
Total motility (%)	41.3 ± 2.0	41.0
Progressive motility (A + B) (%)	28.3 ± 1.9	28.0
Normal morphology (%)	25.3 ± 1.6	28.0
Viability (%)	71.8 ± 1.9	75.7
Apoptosis (%)	15.9 ± 1.4	10.9
Necrosis (%)	12.3 ± 1.0	13.4

**Figure 2:**
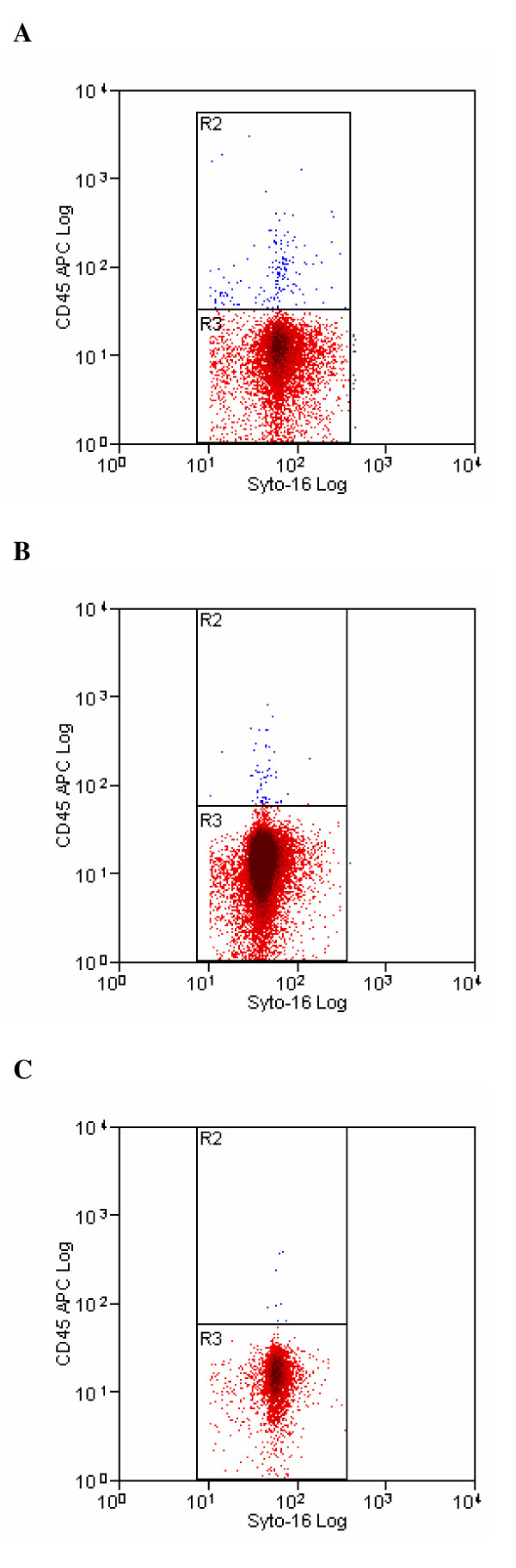
**Pre- (2a) and post-processing (2b, 2c) cytofluorimetric analysis of a semen sample with a low leukocyte concentration**. Cytogram 2a displays the cytofluorimetric analysis of a semen sample containing a low concentration of leukocytes, stained with Syto-16 and CD45 to identify sperm and leukocytes. Region R2 (blue colour) and Region 3 (red colour) represent the number of leukocytes and sperm counted, respectively. Cytograms 2b and 2c displays the cytofluorimetric analysis of the same sample after density-gradient centrifugation and after swim-up technique, respectively.

**Figure 3:**
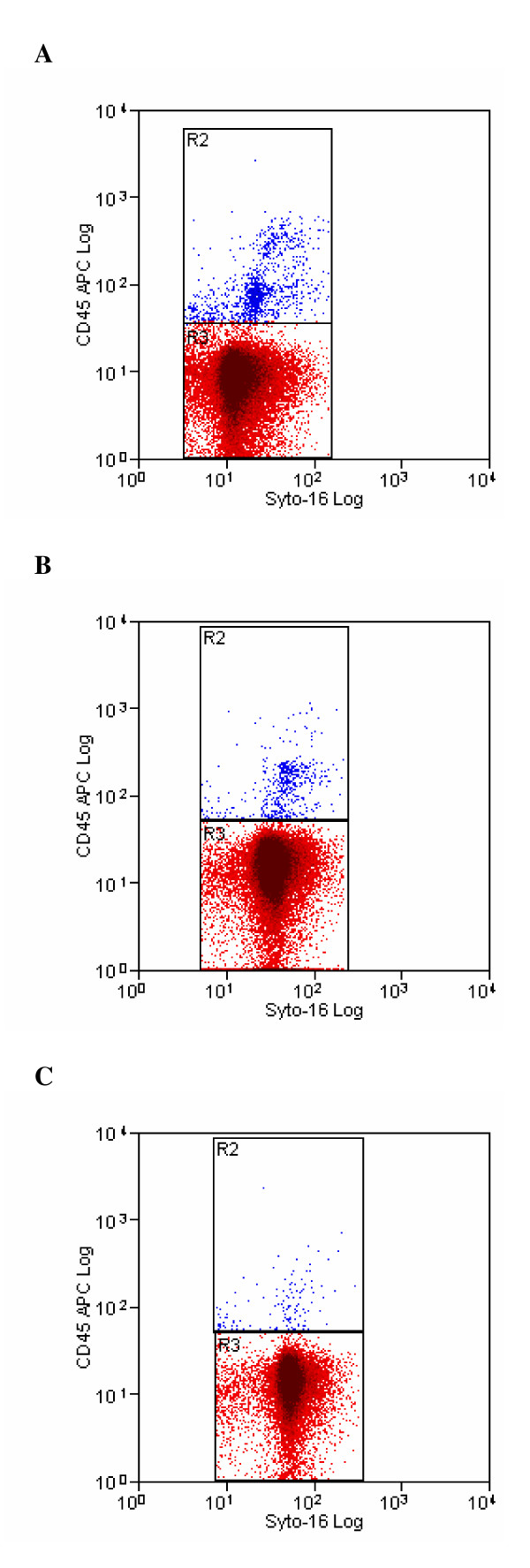
**Pre- (3a) and post-processing (3b, 3c) cytofluorimetric analysis of a semen sample with a moderate leukocyte concentration**. Cytogram 3a displays the cytofluorimetric analysis of a semen sample containing a moderate concentration of leukocytes moderate, stained with Syto-16 and CD45 to identify sperm and leukocytes. Region R2 (blue colour) and Region 3 (red colour) represent the number of leukocytes and sperm counted, respectively. Cytograms 3b and 3c displays the cytofluorimetric analysis of the same sample after density-gradient centrifugation and after swim-up technique, respectively.

**Figure 4:**
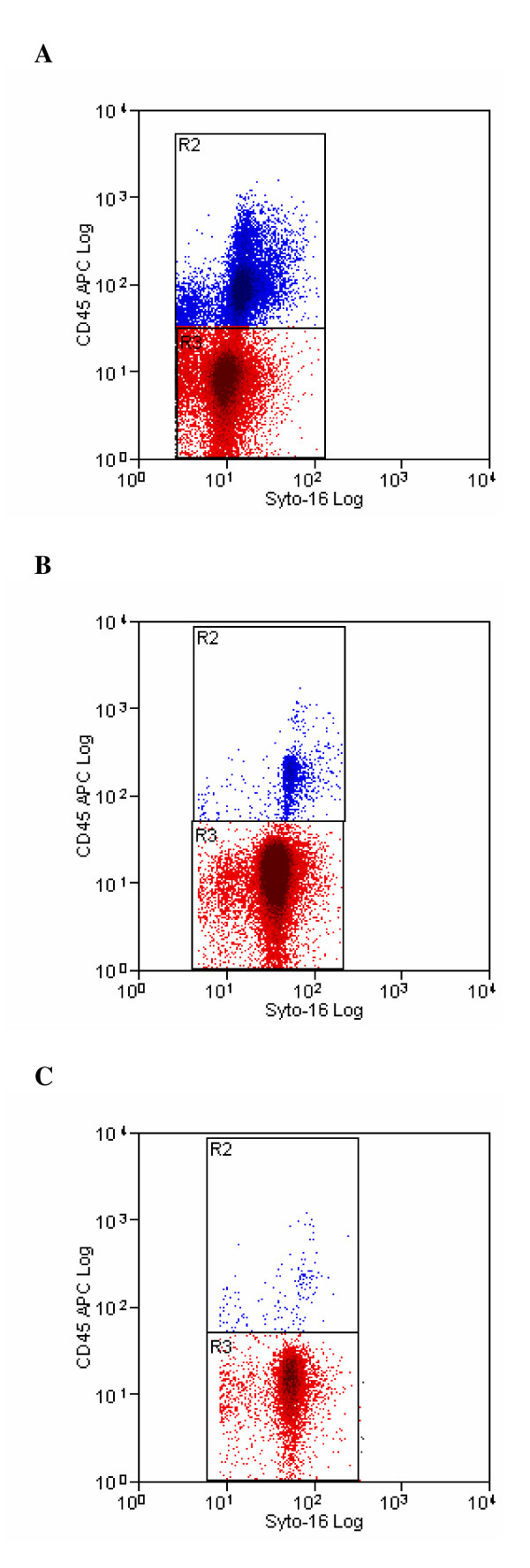
**Pre- (4a) and post-processing (4b, 4c) cytofluorimetric analysis of a semen sample with a high leukocyte concentration**. Cytogram 4a displays the cytofluorimetric analysis of a semen sample containing a high concentration of leukocytes moderate, stained with Syto-16 and CD45 to identify sperm and leukocytes. Region R2 (blue colour) and Region 3 (red colour) represent the number of leukocytes and sperm counted, respectively. Cytograms 4b and 4c displays the cytofluorimetric analysis of the same sample after density-gradient centrifugation and after swim-up technique, respectively.

Flow cytometric analysis of three seminal samples with different concentration of leukocytes, before and after sperm preparation, is shown in Figure 2, Figure 3 and Figure 4. The difference in staining with Syto 16, between the unprocessed and processed sample (Figure 2, Figure 3 and Figure 4), is due to a different uptake of dye by neat and prepared sperm. Furthermore, the unprocessed sample contains epithelial cells, cellular debris, leukocytes, bacteria, etc. that are likewise marked by syto 16 and that are almost completely removed by semen processing.

### Sperm preparation and removal of leukocytes

Compared with the original semen, the concentrations of leukocytes were significantly decreased after both sperm preparation methods, and they were significantly lower after swim-up compared to that after density-gradient centrifugation preparation (Table 2).

**Table 2:** Concentration of seminal leukocytes before and after density-gradient centrifugation and swim-up (n = 69)

	Neat semen	After density-gradient centrifugation	After swim-up	*P value
**Leukocyte Concentration **(10^6^/mL)	0.514 ± 0.120	0.093 ± 0.022	0.018 ± 0.003	< 0.0001

Values are mean ± SEM

* density-gradient centrifugation versus swim-up; Wilcoxon matched pairs test

### Correlation of leukocytes with sperm parameters after semen processing

Leukocytes concentration, either initial or in prepared fractions, was not correlated with sperm optical microscopy parameters (sperm concentration and motility) and flow cytometry parameters (sperm viability, necrosis, and apoptosis) after semen processing. Moreover, the mean percentage of apoptotic sperm after semen processing did not differ significantly between non-leukocytospermic (n = 60) and leukocytospermic patients (n = 9) (8.3 ± 1.0% versus 8.2 ± 1.8%, P = 0.55, after density-gradient centrifugation, and 6.3 ± 1.1% versus 5.0 ± 1.4%, P = 0.8307 after swim-up, respectively). There was no correlation between leukocyte concentration in the ejaculate and sperm recovery rate (Table 3), whereas a significant correlation was found between the concentration of the residual leukocytes in prepared fractions and viable sperm recovery rate (Table 4). This correlation was similar for both semen processing methods.

**Table 3:** Correlation between leukocyte concentration in neat semen and sperm recovery rate (n = 69).

	Total motile sperm recovery rate		Progressive motile sperm recovery rate		Viable sperm recovery rate	
	*r*	*p*	*r*	*P*	*r*	*p*
**DENSITY GRADIENT CENTRIFUGATION**						
Leukocyte Concentration (neat semen)	0.1348	NS	0.1286	NS	0.1478	NS
**SWIM-UP**						
Leukocyte Concentration (neat semen)	-0.1660	NS	-0.0996	NS	-0.0227	NS

r indicates the Spearman's correlation coefficient.

**Table 4:** Correlation between residual leukocytes after sperm preparation and sperm recovery rate (n = 69).

	Total motile sperm recovery rate		Progressive motile sperm recovery rate		Viable sperm recovery rate	
	*r*	*p*	*r*	*p*	*r*	*p*
Leukocyte Concentration (after Density gradient centrifugation)	0.1724	NS	0.1394	NS	0.2871	< 0.05
Leukocyte Concentration (after Swim-up)	0.0130	NS	0.0044	NS	0.2888	< 0.05

r indicates the Spearman's correlation coefficient.

### Peroxidase test and flow cytometry

There was a significant correlation between the total leukocyte count obtained with peroxidase test and that obtained by flow cytometric method (r = 0.5614; P < 0.0001). However, when the same analysis was performed for the groups, a significant correlation was found only in the leukocytospermic group (leukocyte concentration ≥ 1 × 10^6^/mL) (Table 5). The concordance between the two methods for the diagnosis of leukocytospermia is shown in Table 6. The overall concordance was 88,9%, with a Cohen's kappa value of 0.640 (95% CI 0.462 - 0.818). The sensitivity and specificity of Peroxidase test were 68.0% (95% CI 46.5 - 85.0) and 94.1% (95% CI 87.5 - 97.8), respectively.

**Table 5:** Correlation between leukocytes concentration assessed by Optical Microscopy (Peroxidase test) and Flow Cytometry (monoclonal antibody anti-CD45).

**CD45-positive leukocyte concentration (× 10**^3^**/ml)**	r	*P*
< 100 (n = 25)	0.2669	0.1971
100- < 500 (n = 54)	0.06813	0.6245
500-1000 (n = 22)	0.3855	0.0764
> 1000 (n = 25)	0.7109	< 0.0001

Total (n = 126)	0.5614	< 0.0001

r indicates the Spearman's correlation coefficient.

**Table 6:** Concordance between Flow Cytometry and Optical Microscopy for the detection of leukocytospermia

		Flow Cytometry (monoclonal antibody anti-CD45)		Total
		Positive	Negative	
Optical Microscopy (Peroxidase test)	positive	17	6	23
	negative	8	95	103

Total		25	101	

Cohen's K agreement index = 0.640 (95% CI 0.462 to 0.818)

## Discussion

Leukocytes have been reported to negatively influence sperm-egg fusion in experiments [8] and fertilization rate in both IVF and ICSI cycles [14,30-32]. Antibiotic and anti-inflammatory treatment have been suggested for asymptomatic leukocitospermia, but the efficacy of such therapies remains to be definitely established [33-35]. The optimal technique of sperm preparation for assisted reproduction should eliminate dead sperm and other cells, including leukocytes [1]. A recent meta-analysis showed that there are insufficient data from randomized controlled trials to recommend a specific sperm preparation technique [36]. The evidence available does not provide a clear evidence of benefit between the different methods [36]. In the present study, the concentration of leukocytes was significantly lower both after the swim-up and the density gradient preparation compared to neat semen. The low concentration of leukocytes, detected both in swim-up and density gradient fractions, suggests that both methods allow removing most leukocytes. However, swim-up provides a sperm sample with a lower concentration of leukocytes in comparison with density-gradient centrifugation method. Since washed spermatozoa are more sensible to ROS activity [18,22] and ROS can be produced by even few leukocytes [23], our findings suggest that, for assisted conception, semen samples containing leukocytes should be processed using swim-up method. This conclusion conflicts with recommendations by other Authors[1], that suggest not using swim-up in patients with elevated ROS levels in the ejaculate or with genital tract inflammations, but rather employing more gentle methods, like density gradient centrifugation, glass wool filtration or migration-sedimentation. Theoretically, swim-up procedure can exacerbate iatrogenic sperm oxidative stress more than other sperm preparation methods [6], however all these different methods have never been compared in a randomized clinical trial involving patients with elevated ROS or genital tract inflammations.

In this study, we have evaluated the correlation between sperm apoptotic markers and leukocytospermia. The evaluation of sperm viability, apoptosis and necrosis represents a fundamental assessment of sperm quality, although it does not regard other important aspects of sperm function, such as sperm surface and acrosome proteins. Leukocyte concentration in neat semen does not seem to significantly influence the results of semen processing and apoptosis of prepared sperm. Moreover, leukocyte concentration after semen processing was not correlated with sperm parameters, including sperm apoptosis, or recovery rate of motile sperm, whereas it was positively correlated with recovery rate of viable sperm. This significant positive correlation, although weak, was observed both after gradient density-centrifugation and swim-up preparation. Therefore, casual correlation is to be excluded. On the other hand, it has been observed that leukocytes do not always have a negative effect on semen quality and ART outcome [32]. Our findings may support these data. Washed spermatozoa are deprived of the protective effect of seminal plasma and exposed to any toxic oxygen metabolites generated by contaminating leukocytes [18]. It has also been demonstrated that the spontaneous production of ROS by leukocytes is a major source of oxidative stress in washed sperm preparations and that leukocyte contamination impairs sperm motility [18]. To explain these discrepancies with our results, it can be hypothesized that ROS are unable to induce apoptosis in mature sperm because mature sperm do not have efficient operative mechanisms to undergo apoptotic cell death [37,38] or, alternatively, because mature sperm have the ability to scavenge ROS [39]. Moreover, it should be taken into account that ROS may be produced by sperm themselves [23] and that the spermatozoa leukocyte-free, selected with swim-up or density gradient, still produce ROS [13,40,41]. ROS are also essential for capacitation and fertilization [23]. It has been shown that women who became pregnant have significantly higher ROS levels in the follicular fluid than those who failed to conceive [42]. Therefore, the positive or negative effect of ROS on fertility may be a question of concentration. The ROS might induce a damage only when they exceed a specific threshold [32].

World Health Organization recommends peroxidase staining as the standard method and immunocytochemistry using monoclonal antibodies as the gold standard for the detection of semen leukocytes [24]. Although it has been demonstrated that detection of peroxidase-positive cells is not accurate enough to substitute for the immunocytological method [25,26], in most basic and clinical studies seminal leukocytes are currently detected using peroxidase test. On the other hand, immunocytochemistry is expensive, time consuming and not standardized. A few years ago we have shown that flow cytometry using monoclonal antibodies is a simple and reproducible method for detecting semen leukocytes and that the correlation between peroxidase test and flow cytometry results is good but not absolute [43]. Recently, we have optimised this method using multiparameter flow cytometry analysis [27]. In the present study, through the use of this technology we have shown that at low concentrations of leukocytes the correlation between the two methods is very poor. Hence, considering that the majority of patients are non-leukocytospermic, in many cases peroxidase test provides unreliable results. Moreover, we observed that, although the overall agreement between the flow cytometry and the optical microscopy was good, the sensitivity of peroxidase test for the detection of leukocytospermia was low. In fact, only two-thirds of the patients that were diagnosed as leucocytospermic by flow cytometry method have also been identified as such by the optical microscopy. Therefore, using peroxidase test, a significant percentage of patients are wrongly classified as non-leukocytospermic. On the basis of these findings, peroxidase test should be considered not appropriate for clinical research study. Furthermore, its use as a standard test for detecting semen leukocytes in clinical practice should be reconsidered.

## Conclusion

This is the first study that investigated the relationship between seminal leukocytes and sperm preparation using a multiparameter flow cytometer approach that allows performing a simultaneous, accurate assessment of all parameters considered. Seminal leukocytes are not the main inducers of sperm apoptosis in prepared fractions. Seminal leukocytes do not seem to influence sperm preparation results. However, it should be taken into account that in vitro and in vivo studies suggest that both leukocytes in the ejaculate and leukocyte contamination in washed sperm preparations negatively affect fertilization process. Therefore, the most efficient method in removing seminal leukocytes should be preferred. Finally, peroxidase test should not be used in the studies on the semen pathophysiology.

## Competing interests

The authors declare that they have no competing interests.

## Authors' contributions

GR made substantial contributions to conception and design of the study and to analysis and interpretation of data, and wrote major parts of the manuscript. SP made substantial contributions to conception and design of the study and carried out the flow cytometry analysis. RS made substantial contributions to analysis and interpretation of data and wrote parts of the manuscript. RB carried out the routine semen analysis and the flow cytometry analysis and made substantial contributions to analysis of data. MM made substantial contributions to analysis and interpretation of data and wrote parts of the manuscript. LFT made substantial contributions to interpretation of data and reviewed the manuscript. GP Made substantial contributions to design of the study and carried out the flow cytometry analysis. SG made substantial contributions to interpretation of data and reviewed the manuscript. All authors read and approved the final manuscript.
